# A Spatiotemporal Transcriptome Reveals Stalk Development in Pearl Millet

**DOI:** 10.3390/ijms25189798

**Published:** 2024-09-10

**Authors:** Fei Mao, Lin Luo, Nana Ma, Qi Qu, Hao Chen, Chao Yi, Mengxue Cao, Ensi Shao, Hui Lin, Zhanxi Lin, Fangjie Zhu, Guodong Lu, Dongmei Lin

**Affiliations:** 1 National Engineering Research Center of JUNCAO, Fujian Provincial Key Laboratory of Haixia Applied Plant Systems Biology, Haixia Institute of Science and Technology and College of Juncao Science and Ecology, Fujian Agriculture and Forestry University, Fuzhou 350002, China; 2 College of Life Science, Fujian Agriculture and Forestry University, Fuzhou 350002, China; 3 Key Laboratory of Bio-Pesticides and Chemical Biology, Ministry of Education, Fujian Agriculture and Forestry University, Fuzhou 350002, China

**Keywords:** pearl millet, stalk development, gene expression, transcriptome analysis, genetic improvement

## Abstract

Pearl millet is a major cereal crop that feeds more than 90 million people worldwide in arid and semi-arid regions. The stalk phenotypes of Poaceous grasses are critical for their productivity and stress tolerance; however, the molecular mechanisms governing stalk development in pearl millet remain to be deciphered. In this study, we spatiotemporally measured 19 transcriptomes for stalk internodes of four different early developmental stages. Data analysis of the transcriptomes defined four developmental zones on the stalks and identified 12 specific gene sets with specific expression patterns across the zones. Using weighted gene co-expression network analysis (WGCNA), we found that two co-expression modules together with candidate genes were involved in stalk elongation and the thickening of pearl millet. Among the elongation-related candidate genes, we established by SELEX that a MYB-family transcription factor PMF7G02448 can bind to the promoters of three cell wall synthases genes (*CesAs*). In summary, these findings provide insights into stalk development and offer potential targets for future genetic improvement in pearl millet.

## 1. Introduction

Pearl millet (*Pennisetum glaucum* (L.) R. Br., syn. *Cenchrus americanus* (L.) Morrone), domesticated ~4500 years ago in the Sahelian part of West Africa [1], is a C4 cereal crop highly efficient in biomass production. The global planting area of pearl millet has surpassed 31.2 million hectares, ranking it as the sixth most important cereal crop after rice, wheat, maize, barley, and sorghum. Due to its climatic resilience, pearl millet is preferentially cultivated in arid and semi-arid regions such as sub-Saharan Africa, India, and South Asia [2,3], serving as a source of straw for fodder and fuel, and more importantly, serving as the grain source for more than 90 million farmers in the impoverished area [3]. The average yield of pearl millet remained low (0.83 tons per hectare) compared to its genetic potential (3.3 tons per hectare, maximum yield), and this is because it is frequently cultivated in marginal land agricultural systems with a low level of commercial input and little rainfall [4]. Therefore, breeding new varieties of pearl millet with improved productivity and stress tolerance is currently the major challenge.

Improvement in productivity and stress tolerance can both benefit from engineering the phenotypes of stalk. For example, the tillering number of rice can determine its panicle number and subsequently the yield [5], and modulating the lignin content in rice stalk has increased its drought resistance [6]. Moreover, lignocellulose in the stalk of Poaceous grasses is critical for its quality as fodder [7] or industrial raw materials [8]. A reduced lignin content or an increased cellulose content have been reported to promote the feed quality [9]. As observed for maize and elephant grass, the stalk of Poaceous grasses consists of multiple nodes and internodes that grow in an asynchronous manner [10,11,12]. The stalks of Poaceous grasses are also well-known for their fast elongation, which is an integrated output of cell division, cell wall synthesis, and vascular bundle formation [11,13]. However, the molecular mechanisms of stalk development in pearl millet are yet to be explored.

To study the physiology of pearl millet, several recent advances have relied on transcriptomics to elucidate how pearl millet responds to drought [14,15], salt [16,17], and heat [18,19]. The increasing quality and annotation of the genome of pearl millet [3,18] have also eased transcriptomic analysis by avoiding steps of unigene assembly. Therefore, this study aims to measure the transcriptome, capture the dynamics of the transcriptional network during the stalk development of pearl millet, and, in turn, to resolve the key regulators governing stalk morphogenesis. Here, we spatiotemporally sampled 19 transcriptomes from the stalks of four early developmental stages, whereby different internodes on the stalks were separately measured. Based on gene expression, we classified the internode samples and defined four developmental regions on the stalks. We further measured and integrated the phenotypic data and identified the gene modules associated with stalk elongation and thickening by weighted gene co-expression network analysis (WGCNA). Moreover, a local transcriptional regulatory relationship in the module was validated by SELEX (Systematic Evolution of Ligands by Exponential Enrichment), which can be used to ascertain the relative affinity of any transcription factor to any DNA sequence [20]. The generated data and identified genes provide a valuable resource for understanding the stalk development of pearl millet. And it not only deepens our understanding of pearl millet biology but also identifies potential molecular targets for genetic improvement strategies aimed at enhancing the crop’s adaptability and productivity, providing a valuable resource for future genetic improvement efforts.

## 2. Results

### 2.1. Transcriptomic Analyses of Stalks Defined Four Developmental Zones

**Figure 1:**
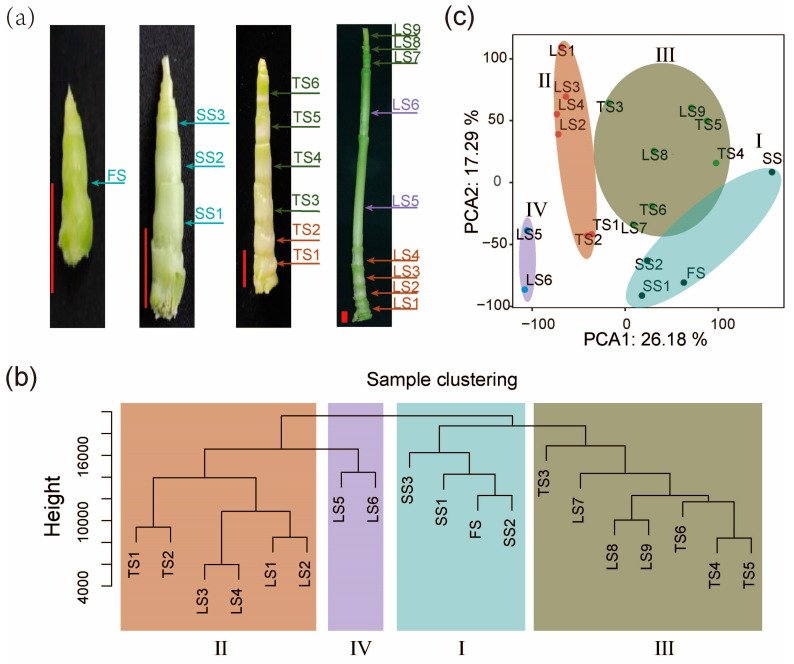
Transcriptomes of internodes reveal four developmental zones of stalks. (**a**) Stalk of pearl millet at four developmental stages. (FS: first stage; SS: second stage; TS: third stage; LS: last stage). Scale: red bars, 1 cm. (**b**) Cluster dendrogram showing the similarities and differences among internode samples based on transcriptomic data. This analysis reveals distinct clusters corresponding to different developmental zones (Zone I–IV), each representing a distinct developmental stage based on gene expression profiles. (**c**) Principal component analysis (PCA) plot indicating the first two principal components (PC1 and PC2), which account for 43.47% of the total variance among samples. The plot classifies the samples into Zone I–IV. Colors represent different developmental zones: Zone I, Zone II, Zone III, Zone IV.

To understand the dynamics of gene expression during the stalk elongation of pearl millet, we collected all internodes for stalks of four early developmental stages (FS: first stage; SS: second stage; TS: third stage; LS: last stage). While different internodes on the same stalk provide spatial resolution, the four time points provide temporal resolution (Figure 1a). Two biological replicates, each of which consisted of pooled samples from at least three plants, were set up for all 19 groups corresponding to individual internodes. We obtained more than 20 million uniquely (~80%) mapped reads on average per RNA-seq sample for subsequent analysis (Table S1), and only the uniquely mapped reads were employed to calculate the normalized gene expression level as transcripts per million (TPM). High reproducibility of the sequence data was observed (r > 0.97) between the biological replicates (Figure S1).

To gain insights into the dynamic transcriptome of pearl millet stalk development, we performed hierarchical clustering (Figure 1b) and principal component analysis (PCA) (Figure 1c). The first two principal components (PCs) of PCA collectively explained 43.47% of the total sample variance in the transcriptomic data. Through PCA and hierarchical clustering, these high-density internode transcriptomes were divided into four clusters, which correspond to four developmental zones (Zone I–Zone IV) on the stalk. Zone I includes four internodes of the earliest (FS and SS) developmental stage. Zone II includes six internodes located at the basis of the stalk in the TS and LS stages. Zone III includes seven internodes from the upper end of the stalk in the TS and LS stages. Zone IV includes the two fast-growing and longest internodes in the middle of the LS-stage stalk (Figure 1b,c).

### 2.2. Gene Clusters Associated with Different Developmental Zone

**Figure 2:**
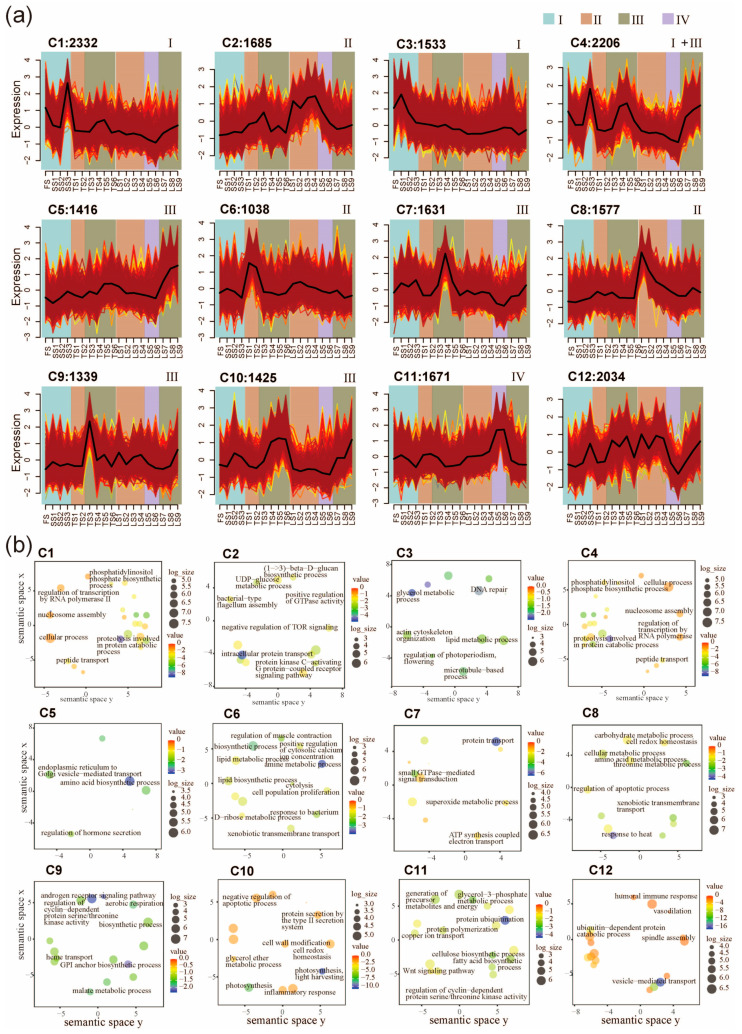
Spatiotemporal pattern of gene expressions and functional enrichment analysis. (**a**) Expression patterns of 19,887 high-variation genes (HVGs) in pearl millet internode samples, classified into 12 clusters (C1–C12) using the fuzzy c-means clustering algorithm. Twelve clusters (C1–C12) were defined. Internodes belonging to different developmental zones are indicated with the background colors. Colored lines represent the gene expression profiles across different internode samples within each cluster. (**b**) Enrichment of functional categories of the twelve fuzzy c-means clusters.

After the sample classification, we next classified genes according to their expression across all internode samples. The expression patterns of all 19,887 high variation genes (HVGs) were clustered into twelve co-expression clusters (C1–C12) using the fuzzy c-means clustering algorithm (Figure 2a and Figure S2, and Table S2). We next performed Gene Ontology (GO) enrichment analysis and dimensional reduction to annotate the potential functions of these clusters (Figure 2b). The results show that C1 (2332 genes) and C3 (1533 genes) contain HVGs that are preferentially expressed in Zone I (Figure 2a). The C1 HVGs are involved in cellular protein metabolic process, nucleosome assembly, translational elongation, and peptide transport (Figure 2b). The C3 HVGs are involved DNA repair and lipid metabolic processes (Figure 2a). Therefore, the C3 HVGs might be involved in rapid cell proliferation because they are mainly expressed at FS, SS1, SS2, and SS3 (Figure 2a). C2, C6, and C8 exhibited peak gene expression in the division zone (Zone II). C6 contained 1038 HVGs associated with response to bacterium, regulation of muscle contraction, and a lipid biosynthetic process. C8 included 1577 HVGs associated with response to heat, regulation of apoptotic process, threonine metabolic process, and xenobiotic transmembrane transport (Figure 2b). These genes might be involved in nutrient transport and defense because they are mainly expressed in the basal internodes (TS1, TS2, LS1, and LS2) (Figure 2a). Genes with peak expression in Zone III are represented by three clusters (C5, C7, C9, and C10), which is the zone containing the most dynamically changed genes (Figure 2a). C5 contained 1416 HVGs, which are mainly involved in the regulation of hormone secretion, endoplasmic reticulum to Golgi vesicle-mediated transport, and intracellular protein transport (Figure 2b). C7 contained 1631 HVGs, which are mainly involved in protein transport, small GTPase-mediated signal transduction, superoxide metabolic process, and ATP synthesis coupled electron transport (Figure 2b). C9 contained 1339 HVGs, which are mainly involved in cytoskeletal organization, cellular component organization or biogenesis, movement of a cell or subcellular components, and microtubule-based processes (Figure 2b). C10 contained 1425 HVGs, which are mainly involved in photosynthesis, cell wall modification, protein secretion by the type II secretion system, and cell redox homeostasis (Figure 2b). Expression of C11 genes peaked in Zone IV, which is the zone containing the least dynamically changed genes (Figure 2a). A total of 1671 HVGs in C11 encoded cellulose synthase (UDP-forming) activity, fatty acid biosynthetic process, glycerol-3-phosphate metabolic process, and Wnt signaling pathway (Figure 2b). This result indicates that C11 may be an important period for cell wall elongation, deposition and reorganization during stalk elongation, and differentiation. A total of 2034 HVGs in C12 encoded vesicle-mediated transport, ubiquitin-dependent protein catabolic process, and humoral immune response (Figure 2b). Interestingly, in contrast to C11, the C12 genes are weakly expressed at LS5 and LS6 (Figure 2a).

C4 HVGs (2206) are most extensively expressed among all gene clusters, as their high-level expressions were observed in Zone I and III. For the TS and LS stages, the expression of C4 genes gradually increases towards the top of the stalk (e.g., LS7 < LS8 < LS9). These genes were involved in nucleosome assembly, peptidase activity, and regulation of transcription by RNA polymerase II. The GO terms are similar to C1 (Figure 2a,b). Taken together, these results reveal that major transcriptomic shifts are associated with stalk development.

### 2.3. Identification of TFs Involved in Stalk Elongation and Growth

**Figure 3:**
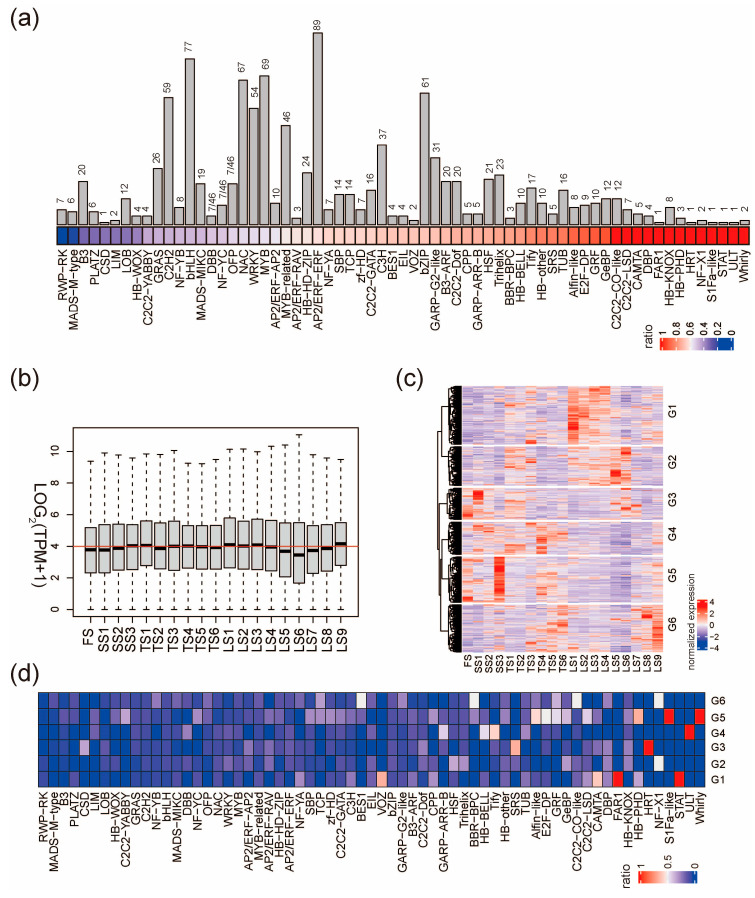
Expression patterns of HVTFs. (**a**) Number (bar plot) and proportion (colored square) of HVTFs in each TF family. For each TF family, the ratio equals the number of HVTFs divided by the number of TFs. (**b**) The expression of all HVTFs in the 19 stalk internodes. The red line indicates that LOG_2_(TPM+1) equals four. (**c**) Clustering heatmap shows six groups of expression patterns of all HVTFs. (**d**) The proportion of HVTFs in each TF family in the 6 groups defined in (**c**).

Transcription factors (TFs) are important “hub genes” in transcriptional networks; therefore, we next profiled their expression patterns during the early stages of stalk development. The HVGs contain 1084 transcription factors (HVTFs), accounting for 56.1% of the 1933 TFs in pearl millet. The HVTFs included 65 transcription factor families (Figure 3a and Figure S3a). Whirly, ULT, and STAT families had the highest ratio of HVTFs, suggesting that TF members of these families could be more relative to stalk development. Consistently, the flowering-related TF families such as RWP-RK and MADS-M-type have the lowest ration of HVTFs. However, for large transcription factor families, it is still possible that only a few members are involved in stalk development, such as the families of AP2/ERF-ERF, bHLH, MYB, WRKY, and NAC (Figure 3a).

The expression levels of all HVTFs were similar across the 19 internodes, except that LS5, LS6, and LS7 have relatively low expression levels (Figure 3b); however, the expression of individual HVTFs shows great variation across the internodes. HVTFs were classified into six groups (G1–G6) according to their expression pattern (Figure 3c). In G1, HVTFs were highly expressed in LS1–LS4 (zone III); in G2, HVTFs were highly expressed in LS5 and LS6 (zone III); in G5, HVTFs were highly expressed specifically in SS3 and least expressed in LS5 and LS6; in G6, HVTFs were highly expressed in LS8 and LS9 (Figure 3c). We next examined the overrepresented TF families in G1–G6 HVTFs and found that members from families such as GeBP and Alfin-like are enriched in specific groups (Figure 3d and Figure S3b,c). Taken together, stalk development requires the time-specific and space-specific expression of a number of key transcription factors.

### 2.4. WGCNA Identifies Interrelated Functional Modules and Hub Genes

**Figure 4:**
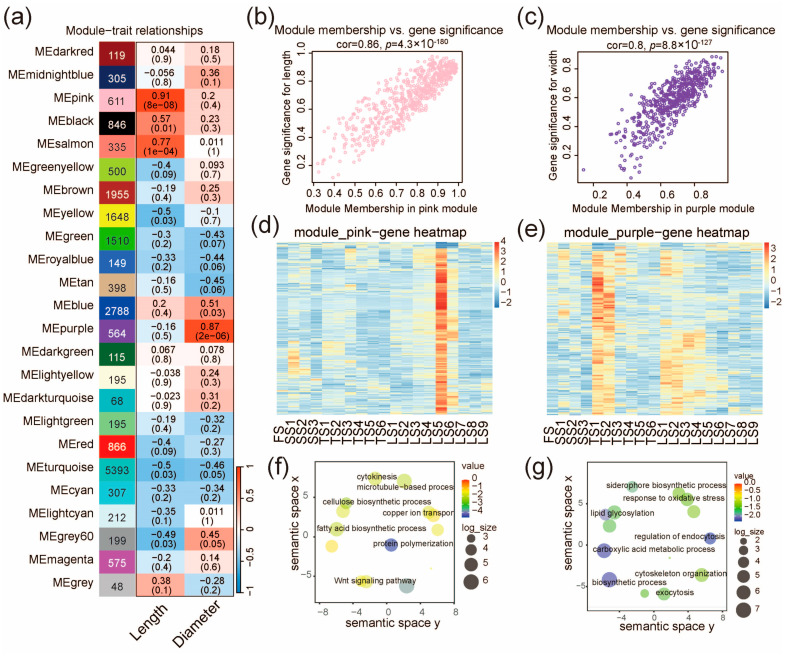
Co-expression network analysis identifies gene modules related to stalk length and diameter. (**a**) Gene modules and their correlation with stalk length and diameter. The number of genes, the correlation coefficient, and the *p* value of the correlation (in brackets) are indicated for each module. The correlation coefficient between the module and trait is shown in red for positive correlations (ranging from 0 to 1) and blue for negative correlations (ranging from −1 to 0). (**b**) Scatter plot for genes in the pink module, illustrating the correlation between their module membership and their effect significance on stalk length. (**c**) Scatter plot for genes in the purple module, illustrating the correlation between their module membership and their effect significance on stalk diameter. (**d**,**e**) The expression profile of all genes in the pink module (**d**) and the purple module (**e**). The color scales represents the Z score of expression level. (**f**,**g**) Enrichment of functional categories for genes in the pink module (**f**) and the purple module (**g**).

To identify gene modules correlated with stalk phenotypes in pearl millet, we conducted WGCNA with a scale independence > 0.85 and a connectivity < 100 as the criteria, and a soft threshold power of 9 (Figure 4 and Figure S4a,b, see also the Methods). In total, we identified 25 gene modules (different colors in Figure 4), and the module eigengene (ME) was examined for their correlation (cutoff: *p* < 1 × 10^−5^) with the measured phenotypes of the stalks (length and diameter of the internodes). The pink module (611 genes) was identified as significantly correlating with the internode length (*r* = 0.91, *p* = 8 × 10^−8^, Figure 4a). Consistently, the module membership of the 611 genes was also correlated with their significance of effect on stalk length (*r* = 0.86, *p* = 4.3 × 10^−180^, Figure 4b). The expression pattern of the pink module genes is similar to that of C11 (Figure 2a and Figure 4d), which are highly expressed in Zone IV (LS5 and LS6). This is also in agreement with the observations that the lengths of LS5 and LS6 are greater than all other internodes (Figure S4c). We further analyzed the key biological processes enriched for genes in the pink module, and one of the enriched GO terms is the cellulose biosynthetic process (Figure 4f), suggesting the indispensable role of cellulose synthesis in stalk elongation.

In addition, we found that the purple module (564 genes) was correlated with stalk diameter (*r* = 0.87, *p* = 2 × 10^−6^, Figure 4a), and that the module membership of the 564 genes was also correlated with their significance of effect on stalk diameter (*r* = 0.8; *p* = 8.8 × 10^−127^, Figure 4c). The expression trend in the purple module genes gradually decreased from bottom to top (Figure 4e). This is consistent with the fact that the measured diameters of the stalk gradually decreased from bottom to top in the three later stages (SS, TS, LS, Figure S4c). The genes in this module are mainly associated with the regulation of endocytosis, lipid glycosylation, and response to oxidative stress (Figure 4g).

### 2.5. Identification Key Genes Associated with Stalk Elongation in Pearl Millet

**Figure 5:**
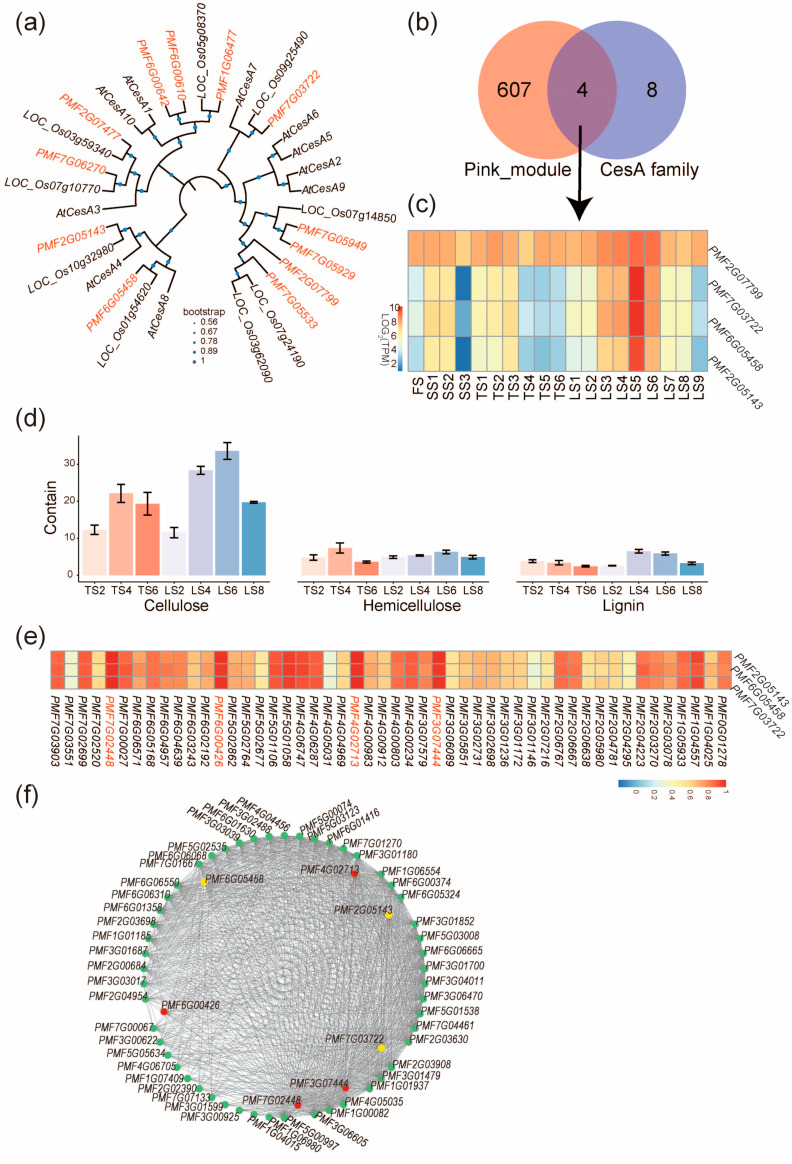
Candidate genes regulating stalk length of pearl millet. (**a**) Phylogenetic tree of CesA family proteins in Arabidopsis (with “At” prefix), rice (with “LOC_Os” prefix), and pearl millet (with “PMF” prefix). (**b**) Venn diagram showing that 4 *PmCesA* genes belong to both the pink module and the *CesA* family genes. (**c**) The expression pattern of the four *PmCesA* genes; note that three of them are expressed more consistently with that of the pink module (Figure 4d). (**d**) Contents of cellulose, hemicellulose, and lignin in the internodes of pearl millet stalks. Error bars represent ± SD (n = 3). (**e**) The correlation between expressions of *PmCesA* and TFs in the internodes of pearl millet stalks. Names of highly correlated TFs are in red. (**f**) Co-expression network showing genes of top-100 highest connectivity in the pink module. Yellow circles represent *PmCesA*, red circles represent the highly correlated TFs in (**e**), and other genes are represented with green circles.

Because cellulose synthesis was extensively reported to affect stalk development [21], we then focused on genes belonging to the pink module and affecting the cellulose biosynthetic process. In the cellulose biosynthetic process, the *CesA* genes encoding the cellulose synthases [22] are pivotal to cellulose synthesis; therefore, we systematically identified *CesA* genes in the genome of pearl millet and found 12 paralogs (Figure 5a). Next, intersecting the pink module with the 12 *PmCesA* genes yielded four genes (Figure 5b), including *PMF2G07799*, *PMF6G05458*, *PMF2G05143*, and *PMF7G03722*, and three of them (*PMF6G05458*, *PMF2G05143*, and *PMF7G03722*) showed an expression pattern more consistent with that of the pink module (Figure 4d and Figure 5c) and were thereby selected for further analyses. Phylogenetic analysis revealed that these three genes, respectively, correspond to a single ortholog in other monocots (*O. sativa*) and dicots (*A. thaliana*) (Figure 5a), suggesting that these three genes were not duplicated in evolution. Their orthologs in Arabidopsis are *AtCesA8*, *AtCesA4*, and *AtCesA7*; all are well-documented genes in cell wall development [23].

To verify the regulation of cellulose synthesis by *PMF6G05458*, *PMF2G05143*, and *PMF7G03722* in stalk growth, we measured the cellulose, hemicellulose, and lignin contents for selected stalk internodes. The highest cellulose content was found in LS6, which agrees with the highest expression of *PMF6G05458*, *PMF2G05143*, and *PMF7G03722* in Zone IV internodes (Figure 5d). The results suggest that *PMF6G05458*, *PMF2G05143*, and *PMF7G03722* potentially regulated cellulose synthesis to control the rapid elongation of the stalks of pearl millet.

To identify the upstream transcription factors (TFs) that regulate the expression of *PMF6G05458*, *PMF2G05143*, and *PMF7G03722*, we examined the 22 transcription factors in the pink module for their expression correlation with *PMF6G05458*, *PMF2G05143*, and *PMF7G03722*. The most correlated TFs (*r* > 0.9) are *PMF7G02448*, *PMF6G00426*, *PMF4G002713*, and *PMF3G07444* (Figure 5e). Next, the hub–gene network of the pink module was constructed to include these four TFs, the three *CesA* genes (*PMF6G05458*, *PMF2G05143*, and *PMF7G03722*), and genes with the top-100 connectivity. From this network, one of the TFs, *PMF7G02448* (MYB family), was found to be associated with the highest connectivity (Figure 5f).

### 2.6. PMF7G02448 (MYB Family) Regulates the Expression of PmCesAs

**Figure 6:**
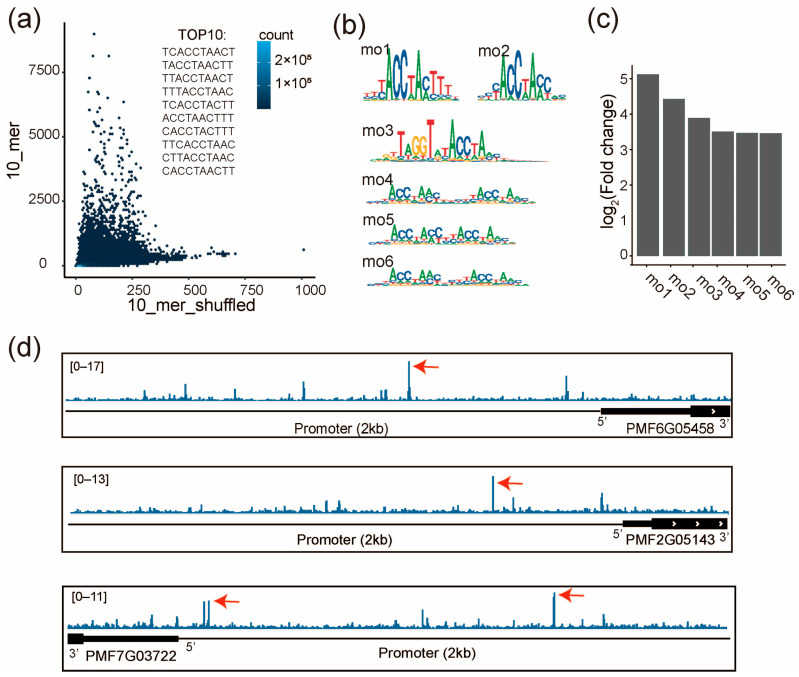
PMF7G02448 (MYB family) directly binds to the promoter of three PmCesA genes. (**a**) Frequencies of all 10-mers are compared between the SELEX library and the same library shuffled. The top 10 most enriched 10-mers are shown. (**b**) The de novo discovered motifs of PMF7G02448. The consensus sequences of mo1-mo6: mo1(YYYACCTAMTTTN), mo2(NYYACCWAMCNN), mo2(NNRKTWGGTRTACCWAMYNN), mo2(NNNACCWAMYNNNNYACCWAMNH), mo2(NNNACCWAMYNYACCWAMNN), mo2(NNNACCWAMYNYYYACCWAMNH). (**c**) Enrichment of each motif in the SELEX library, evaluated by the fold change in motif matches between the SELEX and the shuffled libraries. (**d**) The affinity of genomic sequences to PMF7G02448 is visualized around the three *PmCesA* genes. Red arrows indicate the high affinity sites of PMF7G02448 in the promoter region (transcription start site (TSS) + 2kb). Affinity is defined by the SELEX enrichment scores of 10-mers.

To verify the regulatory role of the MYB-family TF PMF7G02448 on the *PmCesAs*, we performed SELEX for PMF7G02448 to examine its specificity. Two biological replicates agree well with each other (*r* = 0.92, *p* < 2.2 × 10^−16^, Figure S5a). Enrichment analysis show that only a few 10-mers are highly enriched in SELEX, such as TCACCTAACT, TACCTAACTT, and TTACCTAACT, suggesting their high affinity to PMF7G02448 (Figure 6a). Consistently, the de novo motif discovery identified 6 motifs (mo1~mo6, Figure 6b). Fold changes in the motif match between the SELEX library and the shuffled library suggest that the motifs mo1 and mo2 have the highest enrichment (Figure 6c). This demonstrates that PMF7G02448 prefers to bind to both mo1 and mo2. We found the motifs of AtMYB 39 and AtMYB4, which correspond the most to mo1 and mo2, respectively (Figure S5b,c). Furthermore, we discovered that they had similar DNA-binding domains (DBD) by aligning their amino acid sequences (Figure S5d). The results suggested that DBD is a critical sequence for PMF7G02448’s binding motif.

To identify the genomic binding sites of PMF7G02448, we next scored the genome of pearl millet with the SELEX enrichment scores of all 10-mers. The high-score binding sites of PMF7G02448 were identified in the promoter regions of the *PmCesAs* (*PMF6G05458*, *PMF2G05143*, and *PMF7G03722*) (Figure 6d). These results are in agreement with the correlated expression of PMF7G02448 and the three *PmCesAs* (Figure 5e), suggesting that *PMF7G02448* can directly bind to the promoters of the three *CesA* genes to increase their expression.

## 3. Discussion

Pearl millet is a staple crop extensively grown in arid and semi-arid areas [2,3]. To study its stalk development, here, we spatiotemporally resolved the transcriptomes of 19 internodes from four developmental stages of the stalks, and identified the gene modules and gene candidates related to the phenotypes (length and diameter) of stalk internodes.

For internode elongation, previous studies have found that this phenotype is affected by both cell division and cell elongation [24,25], but the two factors contributed differently to different plants. Cell elongation played a major role in rice during the elongation of rice internodes (from 5 to 15 mm), and cells in rice stalks were observed to lengthen threefold [24]. In contrast, in bamboo, the rapid elongation of stalk internodes was found to be explained by cell division at the bottom of the internode and by cell elongation in the top part of the internode [25]. We observed the rapid growth of the single stalk nodes in the middle of pearl millet and presumed that its cell’s undergo rapid growth. At the same time, we also observed that the lower end nodes did not undergo rapid elongation, which may be regulated by a multitude of factors, such as transcription factors, hormones, and other regulatory factors [13].

Cellulose is a major component of plant cell walls [23,26,27]. The *CesA* genes, a highly conserved multigene family encoding the glycosyltransferase enzymes, play an important role in the synthesis of plant cellulose [22,23,27,28,29]. In this study, we observed that the rapid elongation of stalk internodes in pearl millet was associated with cellulose synthesis, as both the expressions of *PmCesA* genes and the cellulose content of Zone IV internodes were up-regulated (Figure 1, Figure 4 and Figure 5). Consistently, the high expression levels of the *CesA* genes were also observed for the rapidly growing tissues of *Linum usitatissimum* [30,31]. While we identified 12 *CesA* family genes in pearl millet, 27 *CesA* genes were found in a closely related species of pearl millet—the tetraploid elephant grass. The increased number of *CesA* genes in elephant grass could have accounted for its higher stalk cellulose content, which exceeds that in maize, wheat, reed, and other plants [32], while the cellulose content in pearl millet is similar to maize (Figure 5d). However, the expression of *CesA* genes (*CesA7-27*) is highest towards the top stalk in elephant grass [11], and this is in contrast to our findings that in pearl millet, the highest cellulose content and *CesA* expression were observed for internodes in the middle of the stalks, thus suggesting that the mechanism underlying stalk elongation can be different, even for closely related species. In this study, we found that the three *PmCesA* genes are co-expressed in the pink module and contribute to the cellulose synthesis during stalk elongation. We have also identified their orthologs in Arabidopsis: *AtCESA4*, *AtCESA7*, and *AtCESA8*. Consistent with the co-expression in pearl millet, the three Arabidopsis orthologs are also co-expressed and involved in the cellulose biosynthesis of the secondary cell wall [33,34,35,36]. As the three *AtCesA* genes are components of the *CesA* complex, we thus infer that the three *PmCesA* genes might also form protein-level complexes during their catalyzes.

The MYB and NAC family TFs have been reported to regulate the expression of *CesA* genes [37,38,39]. For example, previous studies have identified several SCW-specific MYB TFs that can directly regulate the expression of genes encoding lignocellulosic biosynthetic enzymes in dicotyledonous model plants [40,41,42,43]. An electrophoretic mobility shift assay (EMSA) demonstrated that OsMYB58/63 can bind to AC-II (ACCAACC) and SMRE3 (ACCAAAC) sites in the promoter region of OsCesA7 and up-regulate its expression [44]. In pear, PbrMYB24 activates the transcription of the CesA family genes by binding to different cis-elements (AC-I (ACCTACC) element, AC-II element (ACCAACC), and MYB binding site (MBS)) [45]. In agreement with previous findings, in pearl millet, we also found that the TF with the highest expression correlation with the *PmCesA* genes belongs to the MYB family and binds to both AC-I and AC-II elements. Specifically, using SELEX, our study has also illustrated the comprehensive affinity landscape of PMF7G02448 (MYB family) beyond individual promoter sequences. With the enrichment scores obtained in SELEX, we were able to score the whole genome for all the potential binding sites of PMF7G02448. The analysis suggested that the three *PmCesA* genes all harbor high-affinity binding sites of PMF7G02448 in their promoters, and thus are directly regulated by PMF7G02448.

## 4. Materials and Methods

### 4.1. Plant Materials and Sample Collection

Pearl millet (accessions: Tifleaf3) was planted in a greenhouse at a density of one plant per pot (filled charcoal soil mixed with vermiculite in a 3:1 ratio) and grown at a temperature of 28 °C during the light period (16 h) and 22 °C during the dark period (8 h). Stalks at the seedling stages of 25 days (FS: first stage), 35 days (SS: second stage), 56 days (TS: third stage), and 74 days (LS: last stage) were sampled. The internodes under the aerial root were considered underground internodes, and the other internodes were sampled and numbered from the bottom to the top. One sample (stalk internode) was taken at the first stage (FS), three samples at second stage (SS1, SS2, SS3), six samples at the third stage (TS1, TS2, TS3, TS4, TS5, TS6), and nine samples at the last stage (LS1, LS2, LS3, LS4, LS5, LS6, LS7, LS8, LS9) (Figure 1a). All internode samples were then used for RNA extraction and transcriptome analysis. Two biological replicates were collected for every sample.

### 4.2. Determination of the Content of Cellulose, Hemicellulose, and Lignin

The hemicellulose, cellulose, and lignin contents were determined using the traditional Van Soest method [46]. For the chemical measurement, 0.5 g of each internode powder was accurately weighed. NDF (Neutral Detergent Fiber) was obtained by refluxing in a neutral buffered detergent solution for 60 min. Then, the NDF was refluxed in a sulfuric acid solution of 2% hexadecyltrimethylammonium bromide to obtain the ADF (Acid Detergent Fiber) part. Finally, the ADL (Acid Detergent Lignin) part is obtained by treating the ADF with 72% sulfuric acid. NDF represents carbohydrates of cellulose, hemicellulose, and lignin. ADF is composed of cellulose and lignin, while ADL is composed of lignin. All reagents used in this study were of analytical grade.

### 4.3. High-Throughput RNA-Seq and Data Analysis

To measure gene expressions, 0.2 g of each tissue was collected for RNA-seq. The total RNA was extracted from each sample using the RNeasy Plus Mini Kit (Vazyme Biotech, Beijing, China) and cDNA libraries were generated using the NEBNext Ultra RNA Library Prep Kit (New England Biolabs, Ipswich, MA, USA). The quantified libraries were prepared for sequencing on the Illumina HiSeq X-ten sequencing platform. The raw data after removing adaptors and low-quality reads (Q < 20) through Trim Galore v0.6.7 (https://www.bioinformatics.babraham.ac.uk/projects/trim_galore/, accessed on 8 September 2024) were aligned to the Tifleaf3 reference genome [18] using HISAT2 v2.2.1 [47], and gene expression levels were calculated by stringtie v2.2.1 [48] using the default parameters.

### 4.4. Gene Clustering and Gene Family Identification

The high-variation genes (HVGs) of the four periods were detected with a MAD (Median Absolute Deviation) cutoff (>1) and grouped into various clusters using the mfuzz package v2.60.0 [49] with the fuzzy c-means algorithm [49] in R software v3.6.1. OrthoFinder v2.4.5 [50] was used to predict the orthologous relationship of proteins between pearl millet, *Oryza sativa*, and *A. thaliana*. Subsequently, *CesA* genes were identified based on this orthologous relationship. Transcription factors were identified and classified using the iTAK software v1.6 [51].

### 4.5. Construction of Gene Co-Expression Network

Weighted gene co-expression network analysis was performed for 19 internode transcriptomes with the WGCNA package v1.72-5 [52] in R v3.6.1. These genes were filtered with a cutoff (MAD > 1), and then WGCNA was performed to calculate the topological overlap matrix from a pairwise correlation-based adjacency matrix. The neighborhood similarity among genes was calculated, and the gene co-expression modules were identified by average linkage hierarchical clustering. A total of 24 modules were identified using the dynamic hybrid tree cut algorithm and a minimum module size of 50 genes. The networks were visualized with Cytoscape v3.9.1 (http://cytoscape.org/, accessed on 8 September 2024).

### 4.6. Functional Enrichment and Phylogenetic Tree Analysis

GO enrichment analysis of cluster genes and module genes was performed using the Milletdb database [53] (http://milletdb.novogene.com/, accessed on 8 September 2024). The terms with a cutoff (*p* < 0.05) were considered significantly enriched. The biological process terms were visualized using the REVIGO database (http://revigo.irb.hr/, accessed on 8 September 2024) with default parameters to reduce the dimension of GO.

The phylogenetic relationship of the CesA protein families from pearl millet, Oryza sativa, and Arabidopsis thaliana were inferred. Protein sequence alignment was performed with default parameters, and then the resulting sequence was subjected to the maximum likelihood method PhyML to generate a phylogenetic tree in MEGA v11 [54].

### 4.7. High-Throughput SELEX and Data Analysis

The full-length protein coding sequence (CDS) sequence of *PMF7G02448* was inserted into the pIX-Halo vector using the In-Fusion kit (Vazyme, C112, Nanjing, China). The Halo-tagged protein was subsequently expressed using the TnT^®^ SP6 High Yield Wheat Embryo Protein Expression System (Promega, L3261, Fitchburg, WI, USA). The expression system was prepared as follows: 3 µg of reconstituted plasmid was added to 50 µL of the Wheat Embryo Protein Expression Reaction System (Promega, L3261 Fitchburg, WI, USA) and incubated at 25 °C for 2 h to allow for expression of the Halo-tagged fusion protein. The expressed protein was used for SELEX according to the previous report [55,56]. Using 2 µL of fusion protein mixed with 6 µL of SELEX initial library (101 bp, 30 ng/µL) and 30 µL of TCAPT buffer (140 mM KCl, 5 mM NaCl, 1 mM MgCl_2_, 3 µM ZnSO_4_, 100 µM EGTA, 10 mM Tris, pH 8, 0.1% Tween), incubation was performed at 25 °C for 1 h. The DNA–protein complex was then enriched using Halo-tagged magnetic beads (Promega, G7282, Fitchburg, WI, USA) and washed with a HydroSpeed plate washer (Tecan, 30190101, Mennedorf, Switzerland) to remove DNA fragments that had non-specifically bound to the protein. The specifically bound DNA fragments on the beads were then amplified using PCR, and then used as the input library for the next round of SELEX. After four rounds of amplification, the enriched DNA ligands as well as the input library were amplified with paired-end sequencing primers and then sequenced. Sequencing was performed using the Illumina HiSeq X-ten platform. Adapter and low-quality sequences were filtered out using Trim Galore and the paired-end sequences were merged, followed by importing of the sequencing results into Rstudio to remove PCR repeats to obtain clean reads. Enrichments of all 10-mers were computed and de novo motifs were discovered using Autoseed with reported parameters [57,58,59].

## 5. Conclusions

In conclusion, this study provides a comprehensive transcriptomic dataset describing the stalk development of pearl millet. The identification of pivotal modules and genes not only provides insights into the genetic circuits and molecular machinations that control stalk development, but also provides prioritized candidates for future breeding practice. Moving forward, we will prioritize the functional validation of these genes. This will help us better understand the particular roles of these genes in stalk development, directing the development of novel pearl millet varieties that have improved adaptability and production.

## Data Availability

All sequencing data generated from this study have been deposited into the NCBI GEO database under the accession number GSE268902 (https://www.ncbi.nlm.nih.gov/geo/query/acc.cgi?acc=GSE268902, Review token: snmxosaenxgjlon, accessed on 8 September 2024). The sequence data utilized in this study can be found in the Supplementary Materials.

## Supplementary Materials

The following supporting information can be downloaded at https://www.mdpi.com/article/10.3390/ijms25189798/s1.
