# Supplementary material for: A Spatiotemporal Transcriptome Reveals Stalk Development in Pearl Millet
Source: Int J Mol Sci. 2024 Sep 10;25(18):9798. doi: 10.3390/ijms25189798 (PMC11432187; doi:10.3390/ijms25189798)
Supplement: Supplementary file 1 [file ijms-25-09798-s001.zip › Supplementary_files_20240910/20240910sup_infor_fig.docx]

**A Spatiotemporal Transcriptome Reveals Stalk Development in Pearl Millet**

Fei Mao^1,#^, Lin Luo^1,#^, Nana Ma^2^, Qi Qu^1^, Hao Chen^1^, Chao Yi^1^, Mengxue Cao^1^, Ensi Shao^1^, Hui Lin^1^, Zhanxi Lin^1^, Fangjie Zhu^1^, Guodong Lu^3,^, Dongmei Lin^1,*^

^1^ National Engineering Research Center of JUNCAO, Haixia Institute of Science and Technology and College of Juncao Science and Ecology, Fujian Provincial Key laboratory of Haixia applied plant systems biology, Fujian Agriculture and Forestry University, Fuzhou 350002, Fujian, China

^2^ College of Life Science, Fujian Agriculture and Forestry University, Fuzhou 350002, Fujian, China

^3^ Key Laboratory of Bio-Pesticides and Chemical Biology, Ministry of Education, Fujian Agriculture and Forestry University, Fuzhou 350002, China

maofei@fafu.edu.cn (F.M.); 2210514017@fafu.edu.cn (L.L.); manana2014@126.com (N.-N M.); 52362043017@fafu.edu.cn (Q.Q); chenhao19970402@163.com (H.C.); yichao0722@163.com (C.Y.); mxcao0925@163.com (M.-X.C.); es776@fafu.edu.cn (E.-S.S.); ljuncao@163.com (L.H); lzxjuncao@163.com (Z.-X.L.); [fjzhu@fafu.edu.cn](mailto:fjzhu@fafu.edu.cn) (F.-J.Z.)

^#^These authors contributed equally to this work and should be considered co-first authors

*Correspondence: lgd@fafu.edu.cn; lindongmei@fafu.edu.cn

### Supplementary Figures


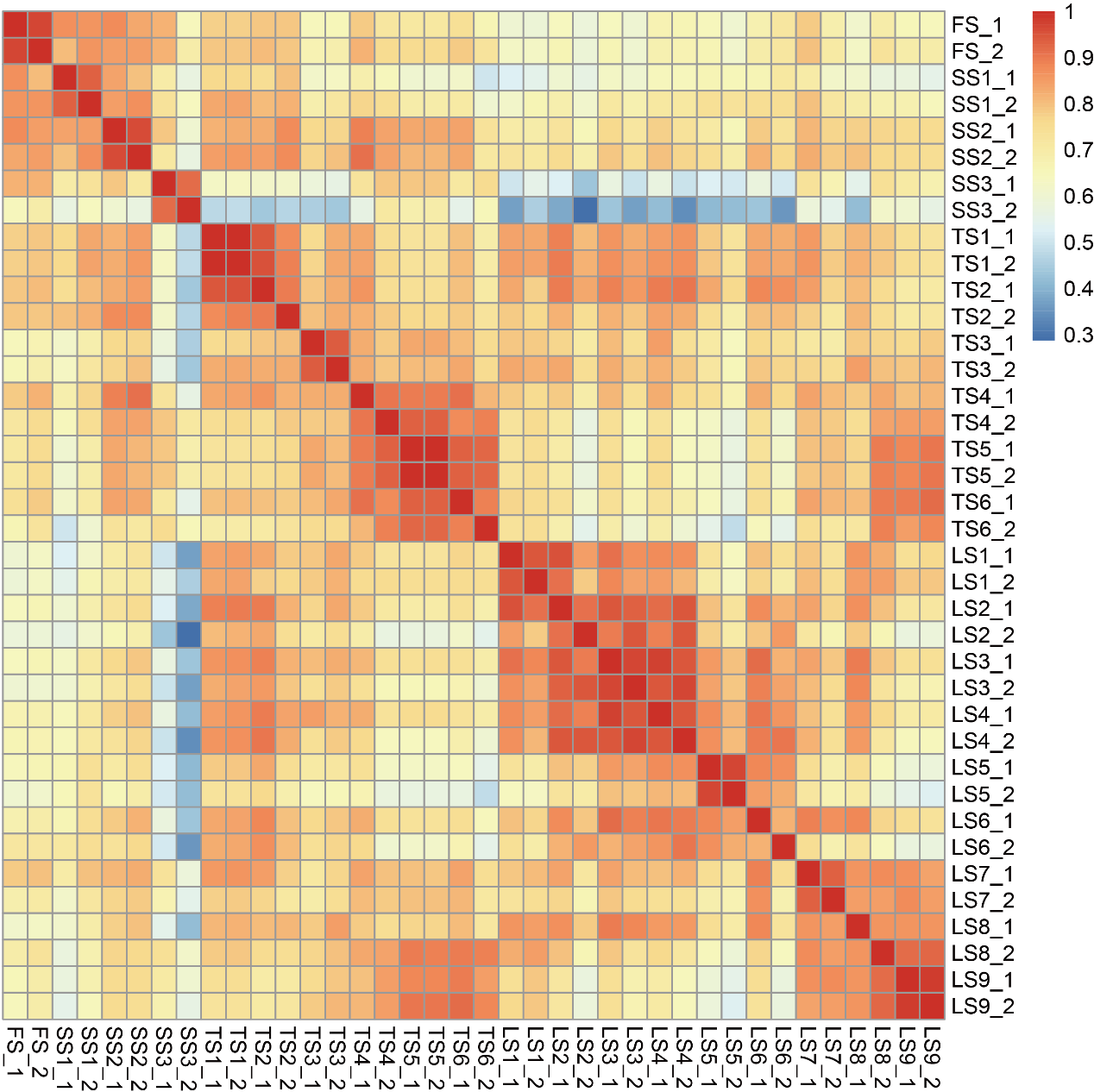


**Figure S1** Heatmap displaying the correlation between all 19 transcriptomic samples (two biological replicates for each sample).


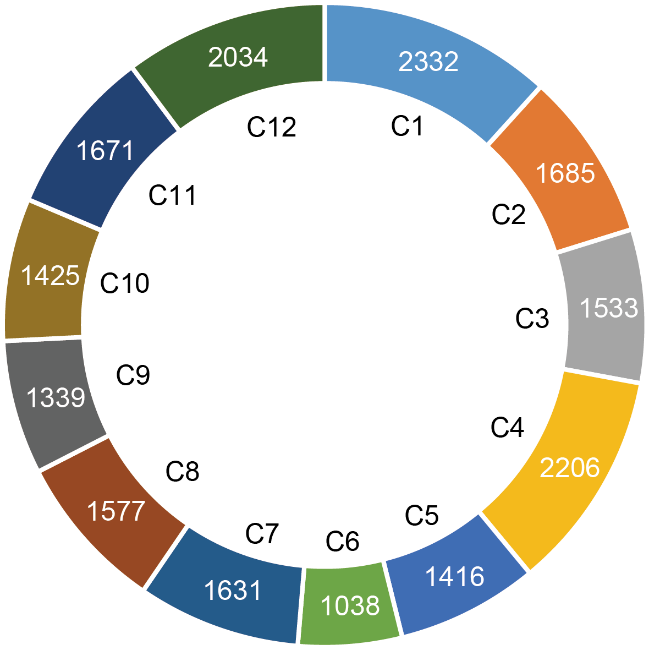


**Figure S2** Pie chart showing the proportion of genes in the twelve clusters (C1-C12s).


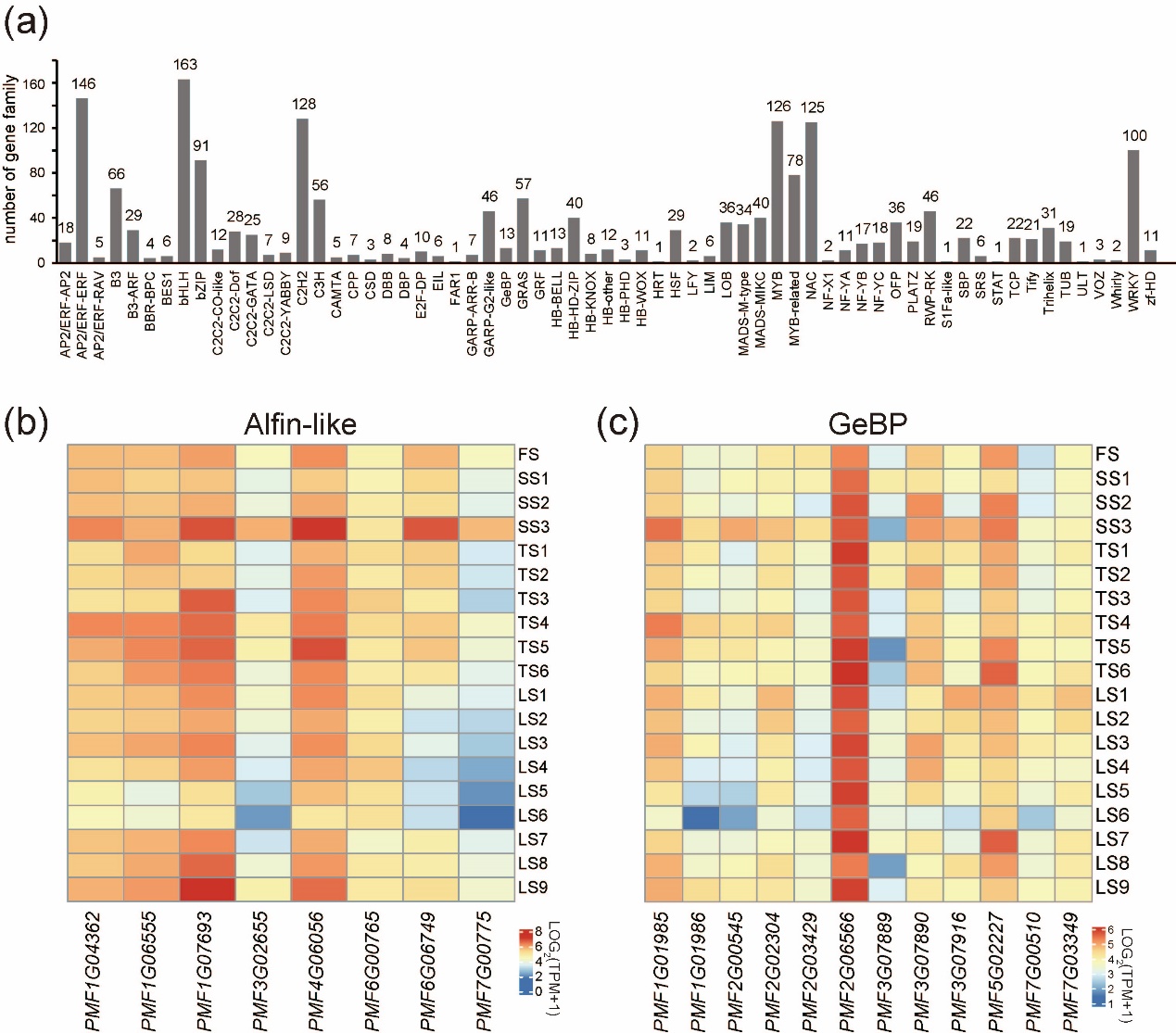


**Figure S3** Transcription factor families and Expression of two key families. (a) Bar plot showing the numbers of pearl millet TFs in each TF family. (b-c) Heatmap showing the expression profile of TFs in the Alfin-like (b) and GeBP (c) families.


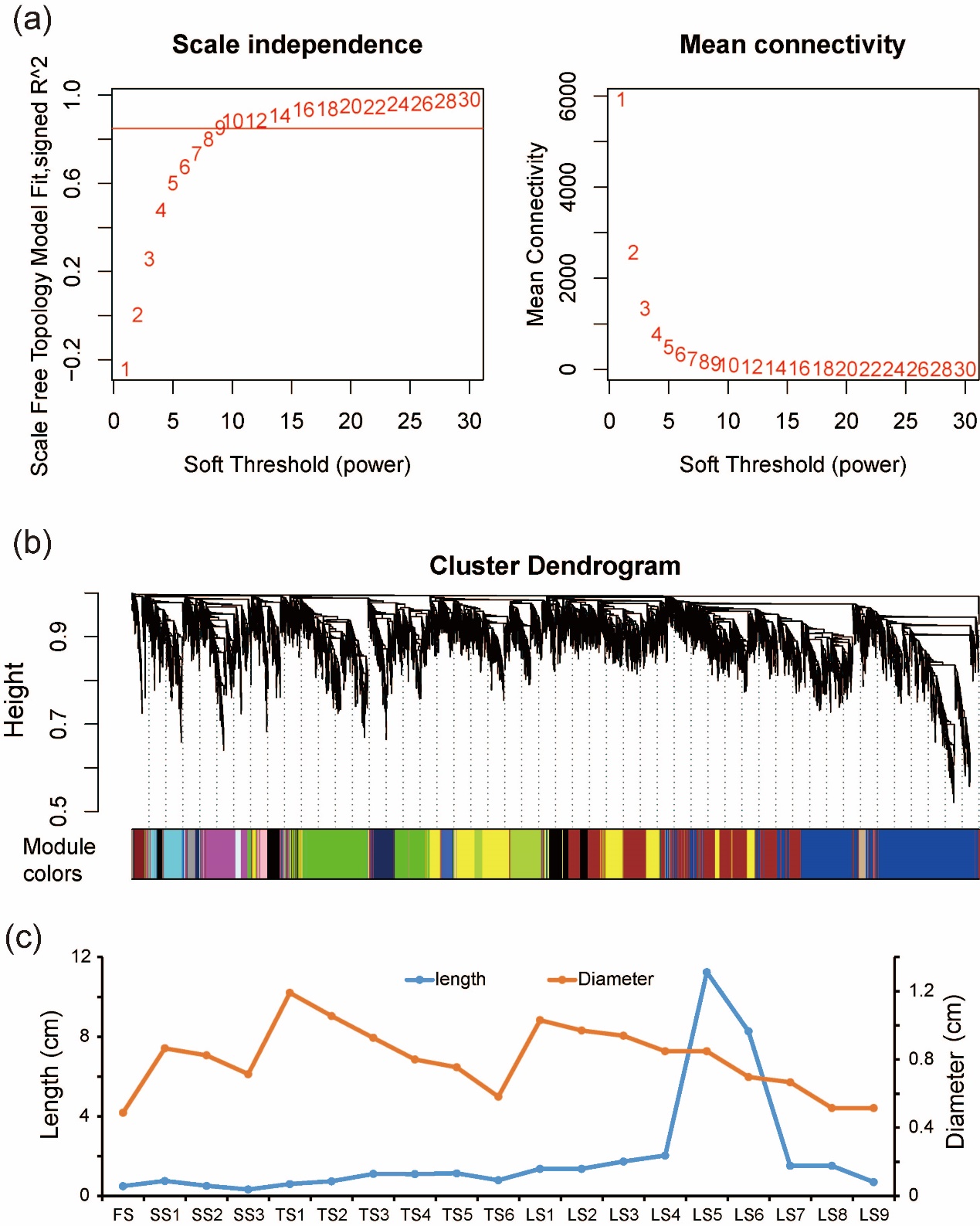


**Figure S4** (a) Scale independence (left) and mean connectivity (right) of the co-expression network at different soft-threshold powers. The left panel displays the correlation of the soft threshold with the scale-free fit index. The right panel displays the influence of soft-threshold power on mean connectivity. (b) The hierarchical tree showing the co-expression modules identified by WGCNA. Each ‘leaf’ (short vertical line) corresponds to an individual gene. The major tree branches constitute 25 modules labelled with different colors. (c) The length and diameter of each internode in the stalks of pearl millet.


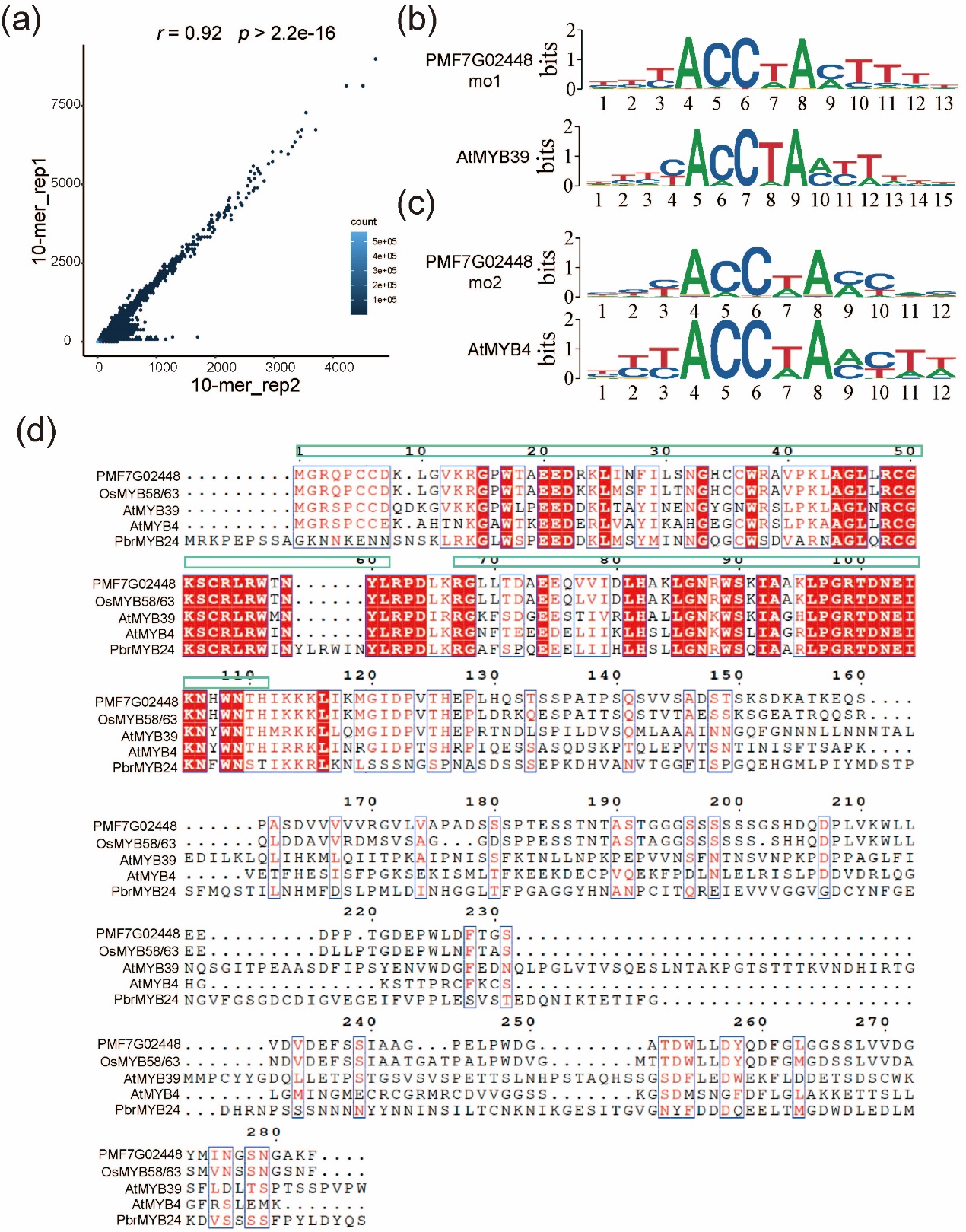


**Figure S5** (a) Correlation between the two biological replicates of SELEX, each dot illustrated the count of each 10-mer in the two libraries. (b-c) Motif models of mo1 (b) and mo2 (c) are consistent with the best previously described motifs. (d) The alignment of the amino acid sequences of PMF7G02448, OsMYB58/63, AtMYB39, AtMYB4, and PbrMYB24 was generated using ESPript 3.0 (https://espript.ibcp.fr/ESPript/ESPript/index.php). The green box indicated DNA binding domain. Os, *Oryza sativa*; At, *Arabidopsis thaliana*; Ptr, *Populus trichocarpa*.
